# Vancomycin-Biomineralized Gold Nanoflowers for In Vitro Photothermal Antibacterial and Antitumor Applications

**DOI:** 10.3390/cells15080680

**Published:** 2026-04-13

**Authors:** Hongying Li, Jinfeng He, Qingtao Zeng, Zhiwei Liu, Haiyan Xiao, Xiaoyu Zhang, Longgang Wang

**Affiliations:** State Key Laboratory of Metastable Materials Science and Technology, Hebei Key Laboratory of Nano-biotechnology, Hebei Key Laboratory of Applied Chemistry, Yanshan University, Qinhuangdao 066004, China

**Keywords:** gold nanoflowers, vancomycin, photothermal therapy, antibacterial, antitumor

## Abstract

**Highlights:**

**What are the main findings?**

- Vancomycin-biomineralized gold nanoflowers were prepared by biomineralization strategy.
- Vancomycin-biomineralized gold nanoflowers exhibited high photothermal conversion efficiency (34.94%) and strong inhibition against *S. aureus* and A549 cells under near-infrared light irradiation.

**What are the implications of the main findings?**

- Vancomycin has both the functions of a biological template, which is good for the formation of vancomycin-biomineralized gold nanoflowers.
- This study developed an efficient photothermal treatment platform, providing a feasible solution for the inhibition of bacteria and tumor cells.

**Abstract:**

Photothermal therapy is a highly promising non-invasive treatment strategy, but its clinical application is still limited by issues such as insufficient light-to-heat conversion efficiency and potential biological toxicity. To address these challenges, this study employed a biomineralization strategy to synthesize gold nanoflowers (Van@Au_4_ NFs) using vancomycin as a template. The synthesized Van@Au_4_ NFs exhibited a uniform flower-like morphology with a hydrodynamic diameter of approximately 122 nm. Under 808 nm laser irradiation, this material demonstrated excellent photothermal properties, with a photothermal conversion efficiency of 34.94%, and remained stable after four cold-hot cycles. The introduction of vancomycin effectively enhanced the colloidal stability and photothermal conversion ability of the nanoflowers. In vitro experiments showed that Van@Au_4_ NFs had an inhibition rate of 90.8% against *Staphylococcus aureus* and 95.18% against A549 tumor cells under near-infrared light irradiation. This study constructed an efficient photothermal agent, providing important experimental evidence for in vitro synergistic photothermal treatment of bacterial infections and tumors.

## 1. Introduction

Cancer and antibiotic-resistant bacterial infections have become major global public health problems, seriously threatening human life and health [1,2]. Currently, the commonly used clinical treatment methods such as surgery, chemotherapy, and radiotherapy have significant limitations. They not only easily induce the emergence of drug resistance but also may cause severe toxic side effects and damage the immune system [3,4,5]. In the field of antibacterial treatment, the abuse of antibiotics further accelerates the spread of antibiotic-resistant bacteria, leading to a continuous decline in treatment effectiveness. Developing new and efficient treatment strategies has become urgent [6,7,8].

Near-infrared photothermal therapy (PTT) is a highly promising emerging physical treatment technology. It uses near-infrared laser (800–1000 nm) to irradiate photothermal agents (PTAs), converting light energy into heat energy to selectively kill tumor cells or pathogens [9,10,11]. Compared with traditional treatment methods, PTT has significant advantages such as strong temporal and spatial controllability and minimal damage to normal tissues [12,13]. Moreover, due to its mechanism of action being physical heat killing, it is less likely to induce bacterial or tumor cells to develop drug resistance, thus having a lower risk of drug resistance [14]. However, the realization of this advantage is highly dependent on the antibacterial microenvironment, laser parameters, and the performance of the photothermal agent, and the problem of insufficient photothermal conversion efficiency still greatly limits its clinical transformation and application promotion [15].

Gold nanoparticles (Au NPs), in particular, have become a highly promising photothermal conversion agent in the field of PTT due to their tunable surface plasmon resonance (SPR) effect [16]. Compared with isotropic spherical gold nanoparticles, anisotropic nanostructures such as star-shaped and flower-shaped nanostructures can generate stronger local electromagnetic field enhancement effects, thereby exhibiting more excellent SPR characteristics and photothermal conversion performance [17,18,19]. However, traditional chemical synthesis methods (such as the sodium citrate reduction method) can only produce spherical gold nanoparticles, making it difficult to precisely control the complex anisotropic morphology; at the same time, the synthesis process often relies on toxic chemical reagents, significantly restricting its potential for biomedical applications [20,21,22].

The biological template method provides an innovative solution to these problems [23,24]. This method uses natural biological molecules as templates to achieve green synthesis of nanomaterials under mild conditions, reducing the use of harmful chemical reagents, and precisely controlling the size, morphology, and structure of nanomaterials, improving their functional characteristics and application value. Vancomycin, as a classic glycopeptide antibiotic, can inhibit cell wall synthesis by specifically binding to the D-alanine-D-alanine (D-Ala-D-Ala) residues of the cell wall precursor of Gram-positive bacteria and induce bacterial death [25,26,27]. It also has good biological recognition ability. Moreover, the molecule of vancomycin contains functional groups such as phenolic hydroxyl and amino groups, with excellent coordination and reduction capabilities, which can serve as an active site for metal ion reduction and nucleation, guiding the directional growth and morphology regulation of inorganic nanomaterials through molecular self-assembly, ultimately preparing structurally uniform and highly performing nanomaterials. Therefore, synthesizing nanoparticles using vancomycin as a biological template has the dual advantages of green synthesis and precise structure control, providing an important strategy for the preparation of new photothermal agents.

Based on this, the current study addresses problems of current PTT photothermal agents, such as limited photothermal conversion efficiency and difficulty in precisely controlling anisotropic morphology. Using vancomycin hydrochloride as a biological template, we constructed gold nanoflowers with high photothermal conversion efficiency, aiming to achieve dual efficient inhibition of bacteria and tumor cells. This study describes the first synthesis of vancomycin-templated gold nanoflowers. (Van@Au_4_ NFs). We systematically characterized their morphology and photothermal properties, and comprehensively evaluated their inhibitory effects on *Staphylococcus aureus*, *Escherichia coli*, and tumor cells in vitro. This study provides an effective solution to improve the photothermal conversion efficiency and is good for the development of a multifunctional therapeutic platform for precision medicine.

## 2. Materials and Methods

### 2.1. Materials

Vancomycin (Van) hydrochloride was purchased from Aladdin Reagent Co., Ltd. (Shanghai, China), chloroauric acid was purchased from Chengdu West Asia Chemical Industry Co., Ltd. (Linyi, China), ascorbic acid was purchased from Tianjin Guangfu Fine Chemical Co., Ltd. (Tianjin, China), and thiazolyl blue (MTT) was purchased from Aladdin Reagent Co., Ltd. (Shanghai, China).

### 2.2. Cell Lines and Bacterial Strains

A549 cells (human lung adenocarcinoma cell line) were obtained from the Cell Bank of the Chinese Academy of Sciences (Shanghai, China). *Escherichia coli* (*E. coli* ATCC 25922) and *Staphylococcus aureus* (*S. aureus* ATCC 6538) were obtained from the laboratory-preserved bacterial stock cultures at Yanshan University.

The A549 cells were cultured in DMEM medium (produced by HyClone Company, Logan, UT, USA), supplemented with 10% fetal bovine serum (FBS, produced by Zhejiang Tianhang Biotechnology Co., Ltd., Hangzhou, China) and 1% penicillin-streptomycin solution (produced by Beijing Labgic Technology Co., Ltd., Beijing, China).

Bacterial culture was conducted using LB liquid medium (peptone 10 g/L, yeast extract 5 g/L, NaCl 10 g/L, made up with deionized water, sterilized at 121 °C under high-pressure steam for 20 min); for solid culture, LB agar medium (with 1.5% agar powder added, the sterilization conditions were the same as before) was used. A single colony was picked and inoculated into 5 mL of LB liquid medium, then placed in an aerobic shaking incubator at 37 °C and 180 rpm for 12 to 16 h to reach the logarithmic growth phase (OD_600_ ≈ 0.6–0.8). Subsequently, it was diluted to the required concentration according to the experimental needs for storage.

### 2.3. Characterization Method

The ultraviolet-visible spectrum (UV-TU1810) is from Shanghai Pu Xie General Co., Ltd. (Shanghai, China), the Fourier transform infrared spectrometer (E55-FRA106) is from Germany Bruker, the X-ray diffractometer (D/max-2500/PC) is from Japan Rigaku, the scanning electron microscope (S-4800) and the transmission electron microscope are from Japan Hitachi, the laser particle size analyzer (Zetasizer Nano-ZS90) is from Malvern (Malvern, UK), the constant temperature mixer (MSC-100) is from Hangzhou Aosheng Instrument Co., Ltd. (Hangzhou, China), the enzyme analyzer (SpectraMax M2) is from the United States Thermo (Waltham, MA, USA), and the vacuum freeze-drying machine (FD-1B-50) is from Beijing Boyikang Experimental Instrument Co., Ltd. (Beijing, China).

### 2.4. Preparation of Van@Au_n_ NFs

**Figure 1:**
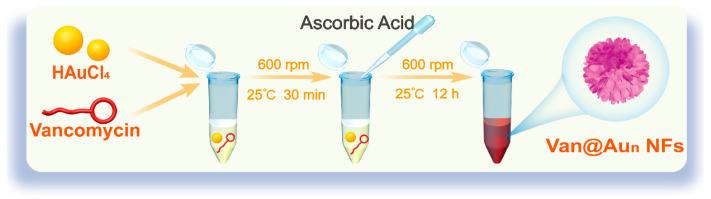
Schematic diagram of the synthesis of vancomycin-biomineralized gold nanoflowers (Van@Au_n_ NFs).

As shown in Figure 1, a total of 800 μL of vancomycin hydrochloride solutions of varying concentrations were mixed with 400 μL of 2 mM chloroauric acid (HAuCl_4_) solution in 2 mL centrifuge tubes. The molar ratios of vancomycin hydrochloride to HAuCl_4_ were 1:0.125, 1:0.25, 1:0.5, 1:2, 1:4, and 1:8, respectively. The mixtures were incubated in a thermomixer (600 rpm, 25 °C) for 30 min. Next, we slowly added 180 μL of freshly prepared 60 mM ascorbic acid (AA) solution dropwise to the incubated solutions. The mixture was gently shaken and then incubated in a thermomixer at 600 rpm and 25 °C for 12 h. The sample was dialyzed against deionized water for 24 h using a dialysis membrane with a molecular weight cutoff (MWCO) of 8–14 kDa. Van@Au_n_ NFs with varying vancomycin hydrochloride ratios (n = 0.125, 0.25, 0.5, 2, 4, and 8) were successfully prepared.

### 2.5. Preparation of Au NPs

Gold nanoparticles (Au NPs) were prepared by adding 500 μL of HAuCl_4_ (2 mM) solution into a 2 mL centrifuge tube. Then, 500 μL of deionized water was added and the mixture was shaken well. Subsequently, 50 μL of freshly prepared 60 mM AA was slowly added dropwise, and the mixture was gently shaken until the solution color changed from pale yellow to uniform red, indicating the successful preparation of Au NPs. The thus-prepared Au NPs solution was used as the control group.

### 2.6. Photothermal Conversion Performance of Van@Au_4_ NFs

A total of 1 mL of Van, Au NPs, and Van@Au_4_ NFs was added to 2 mL centrifuge tubes, respectively. A temperature probe was inserted below the liquid level in the centrifuge tubes. An 808 nm laser with a power of 1.75 W/cm^2^ was used to irradiate the sample solutions. The Van and Au NPs solutions served as the control group. A temperature recorder recorded the solution temperature every 30 s. Lyophilized Van@Au_4_ NFs powder was accurately weighed and redispersed in deionized water to prepare aqueous suspensions with total solid mass concentrations of 37, 74, 148, and 296 μg/mL, respectively. The sample solutions were irradiated with an 808 nm laser at a power of 1.75 W/cm^2^. The temperature changes of the samples were recorded with a temperature recorder over time to observe the temperature rise of the sample solutions at different concentrations under the same laser power density.

At room temperature, the sample solutions were irradiated with an 808 nm laser at a power of 1.75 W/cm^2^. After 10 min, the near-infrared laser was turned off, and the samples were allowed to cool naturally to room temperature. The temperature changes of the samples were recorded with a temperature recorder over time. This process was repeated four times to verify the stability of the samples.

### 2.7. Antibacterial Performance Testing of Van@Au_4_ NFs

A total of 20 mL of culture medium was placed in a small conical flask, to which 1 mL of *Staphylococcus aureus* was added. The flask was shaken to disperse the bacteria, then sealed and incubated on a shaker. Initially, the bacterial concentration was determined using a 150 μL aliquot. The bacteria were diluted by 0×, 2×, 4×, and 8×, respectively, and the OD values at a wavelength of 600 nm were measured using a microplate reader (the OD value closest to 0.2 was identified to determine the bacterial concentration as 2.5 × 10^7^). After dilution to the desired concentration, the bacterial suspension was added to a 96-well plate. The flask was shaken after every five or six wells to ensure homogeneity. From the second to the seventh row, each well was filled with 150 μL of the bacterial suspension. For the preparation of a concentration gradient of the test compounds (Van@Au_4_ NFs, Au NPs, and Van), stock solutions were prepared at concentrations of 0.75 mM, 0.375 mM, 0.187 mM, and 0.093 mM based on gold. Each concentration drug was added in triplicate, with 150 μL added to three wells per row. A 2-fold serial dilution was performed, starting from the second row and continuing through the fifth row. After the addition of all compounds, the initial OD at 600 nm was measured. The inoculated 96-well plate was sealed with a lid and placed in a 37 °C incubator. Bacterial growth was observed and recorded after 48 h of incubation.

### 2.8. In Vitro Antitumor Activity Testing of Van@Au_4_ NFs

A 50 mL centrifuge tube was prepared with 15 mL of culture medium, and then the cell suspension was added. Once the cell suspension was prepared, it was gently shaken to mix well, and 200 μL was added to each well. The cells were then cultured in an incubator. Once the 96-well plates were filled with single cells, different drug concentration gradients were added. Van@Au_4_ NFs exhibited certain aggregation in DMEM. For the laser irradiation group, after 12 h of culture, the cells were irradiated with an 808 nm laser (1.75 W/cm^2^) for 10 min. After another 12 h of culture in a CO_2_ incubator, the liquid in the wells was aspirated and 100 μL (0.5 mg/mL) MTT solution was added to each well. After 4 h of incubation with MTT, crystals were fully formed. The supernatant was removed and 12.5 and 100 μL of dimethyl sulfoxide was added to each well. The plates were shaken at low speed for 10 min to fully dissolve the blue-purple formazan precipitate. The absorbance at a wavelength of 490 nm was then measured using a microplate reader.

## 3. Results

### 3.1. Synthesis and Characterization of Van@Au_4_ NFs

Due to their excellent biocompatibility, peptide-based multifunctional nanosystems have played an important role in the development of biomedicine, particularly in tumor diagnosis and treatment. Vancomycin hydrochloride, with its nine hydroxyl groups, two primary amino groups, and one carboxyl group, possesses excellent hydrophilicity. Furthermore, the amino and hydroxyl groups possess complexing properties, allowing them to partially complex AuCl_4_^−^. The amino acid residues in vancomycin hydrochloride possess reducing properties, allowing them to partially reduce HAuCl_4_ to form small gold particles.

**Figure 2:**
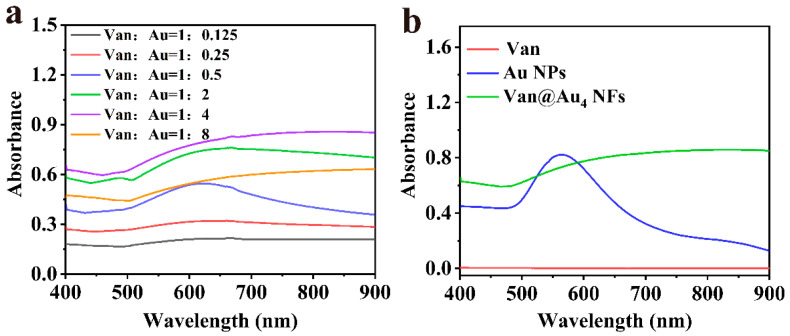
(**a**) UV-visible absorption spectra of Van@Auₙ NFs prepared with different molar ratios of vancomycin (Van) to Au precursors. (**b**) UV-Visible absorption spectra of pure Van, spherical gold nanoparticles (Au NPs), and vancomycin-mineralized gold nanoflower (Van@Au_4_ NFs).

As shown in Figure 2, all Van@Auₙ NFs of all ratios exhibit broad absorption peaks within the 550–900 nm range, which is a typical characteristic of anisotropic gold nanoflowers (rather than spherical gold nanoparticles). As the ratio of vancomycin to HAuCl_4_ increases from 1:0.125 to 1:4, the intensity of the absorption peak at 808 nm gradually increases; when the ratio further increases to 1:8, the absorption peak intensity decreases instead. The absorption peak intensity is the highest when the ratio is 1:4, indicating that the gold nanoflowers generated under this condition have the strongest near-infrared light absorption ability, suggesting the best photothermal conversion efficiency. This phenomenon results from the dual role of vancomycin as a template: it controls the nucleation rate of gold ions through its reducing ability and guides the directional growth of nanoflowers through molecular self-assembly. When the ratio is 1:4, this regulation reaches the optimal balance.

Vancomycin shows no obvious absorption peak within the 400–900 nm range, indicating that it does not possess near-infrared photothermal response capability on its own. Spherical gold nanoparticles (Au NPs) exhibit a typical spherical gold nanoparticle characteristic absorption peak at 563 nm, but there is no significant absorption in the near-infrared region (600–900 nm). This feature is consistent with the absorption behavior of conventional spherical gold nanoparticles prepared by sodium citrate reduction. Van@Au_4_ NFs exhibit a broad and red-shifted characteristic absorption band within the 600–900 nm range, which is the surface plasmon resonance (SPR) effect caused by the anisotropic structure of the gold nanoflowers. Compared with spherical Au NPs, the red shift and broadening of the absorption peak are due to the stronger local electromagnetic field enhancement generated by the complex branching structure of the nanoflowers, which enhances the capture ability of near-infrared light and thereby improves the photothermal conversion efficiency.

**Figure 3:**
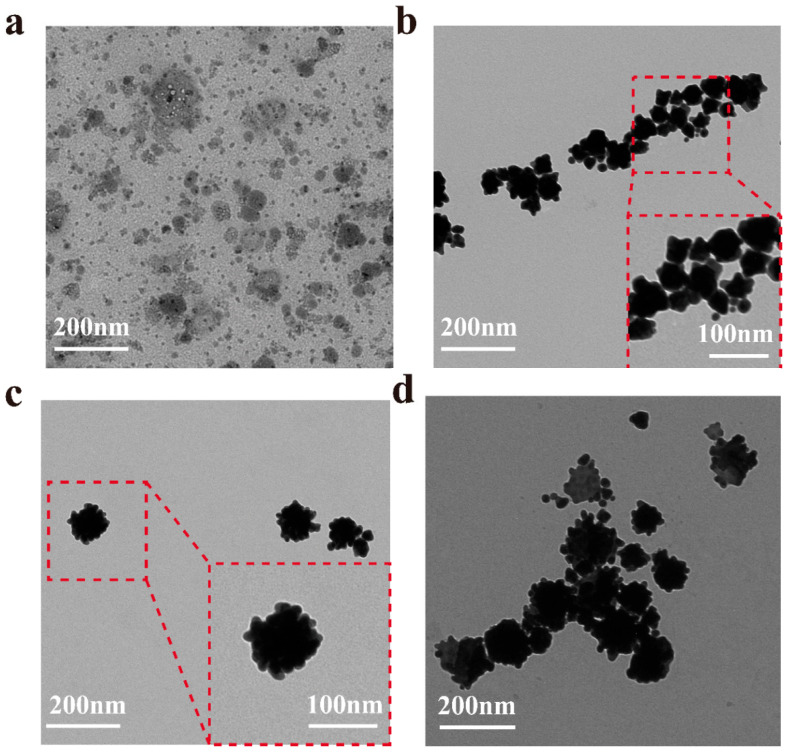
Low-magnification electron microscopy images (scale bar: 200 nm) and high-magnification images of selected areas (scale bar: 100 nm) of Van@Au_4_ NFs at (**a**) 0 h, (**b**) 3 h, (**c**) 12 h, and (**d**) 24 h.

To investigate the effect of reaction time on the morphology and size of nanoparticles, Van@Au_4_ NFs samples with different reaction times were prepared under the same conditions. Figure 3a shows the transmissive sample immediately after the addition of the reducing agent ascorbic acid. Only small particles were observed, with no gold nanoflowers synthesized. Figure 3b shows the transmissive sample after 3 h of reducing agent addition. At this point, the average particle size of the gold nanoflower was 58.9 nm, and its shape was irregular. Figure 3c shows the sample after 12 h of reducing agent addition. At this time, the gold nanoflowers have a particle size of approximately 100 nm and a uniform morphology, demonstrating good dispersion. Figure 3d shows the sample after 24 h of reducing agent addition. At this time, the gold nanoflowers have aggregated. Therefore, Van@Au_4_ NFs have the best morphology.

**Figure 4:**
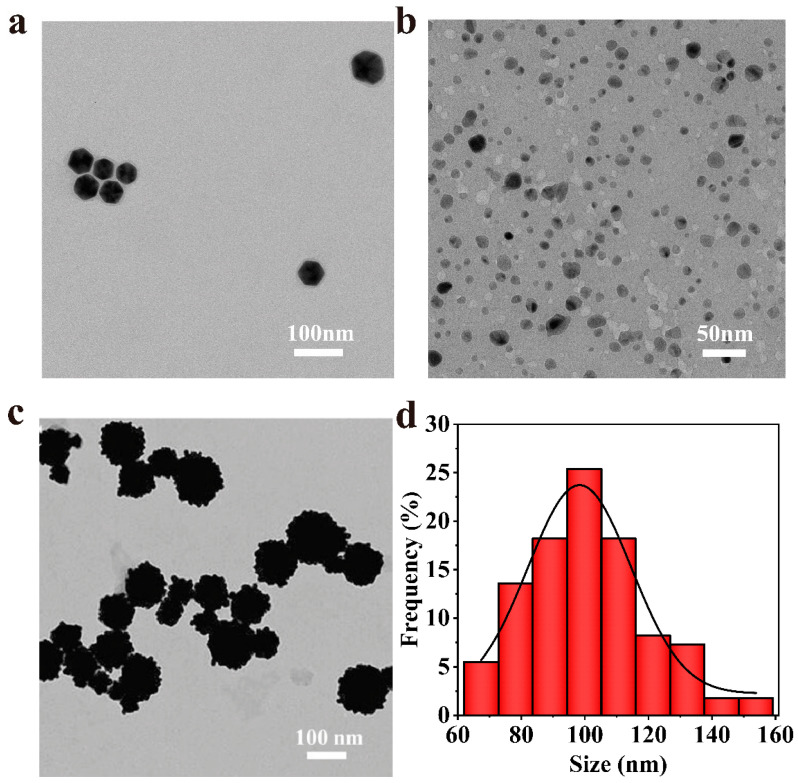
(**a**) TEM image of metal nanoparticles produced by incubation of vancomycin hydrochloride with HAuCl_4_ (scale bar: 100 nm). (**b**) TEM image of metal nanoparticles produced by incubation of ascorbic acid with HAuCl_4_ (scale bar: 50 nm). (**c**) TEM image of Van@Au_4_ NFs produced by incubation of vancomycin hydrochloride with HAuCl_4_ and reduction with ascorbic acid (scale bar: 100 nm). (**d**) Particle size distribution of Van@Au_4_ NFs.

Van@Au_4_ NFs were prepared using a simple green template method. In this reaction system, HAuCl_4_ was used as the precursor, ascorbic acid was used as the reducing agent, and vancomycin hydrochloride was used as the template. Figure 4a shows a TEM image of nanoparticles obtained after incubation of Van and HAuCl_4_ for 12 h. The nanoparticles are hexagonal and uniformly dispersed, with a particle size of 50–60 nm, indicating that the amino acid residues in the template vancomycin have a certain degree of reducing activity. Figure 4b shows Au NPs prepared using HAuCl_4_ and ascorbic acid. The nanoparticles are spherical and have a particle size of approximately 10–20 nm. Figure 4c shows a transmission electron microscope image of Van@Au_4_ NFs prepared by incubating Van and HAuCl_4_ for 30 min, adding the reducing agent (ascorbic acid), and then incubating for an additional 12 h. The gold nanoparticles in Van@Au_4_ NF are larger than 50 nm and uniformly dispersed, with a predominant flower-like shape. This demonstrates that vancomycin serves as a template for the formation of the flower-like Van@Au_4_ NFs. The particle size of gold nanoparticles was further counted. As shown in Figure 4d, the average size of gold nanoparticles in Van@Au_4_ NFs is 102.94 nm.

**Figure 5:**
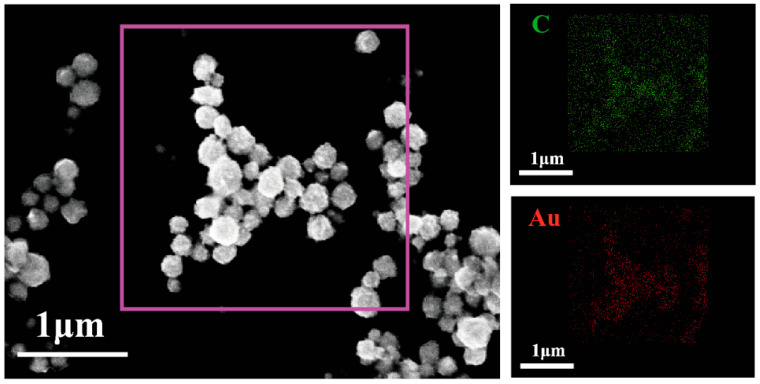
SEM image of Van@Au_4_ NFs (scale bar: 1 μm) and its corresponding C and Au element distribution.

In addition to observing the morphology and size of the nanoparticles using transmission electron microscopy, scanning electron microscopy can also be used to test the morphology and size of Van@Au_4_ NFs and observe the elemental distribution within them. Figure 5 shows the morphology and size of the Van@Au_4_ NFs. As can be seen from the image, the surface of the Van@Au_4_ NFs is uneven and flower-like, with a uniform particle size ranging from 110 to 140 nm, consistent with the particle size observed using transmission electron microscopy. Furthermore, Van@Au_4_ NFs contain Au and C elements. The C is primarily derived from vancomycin hydrochloride, while the Au is derived from chloroauric acid.

**Figure 6:**
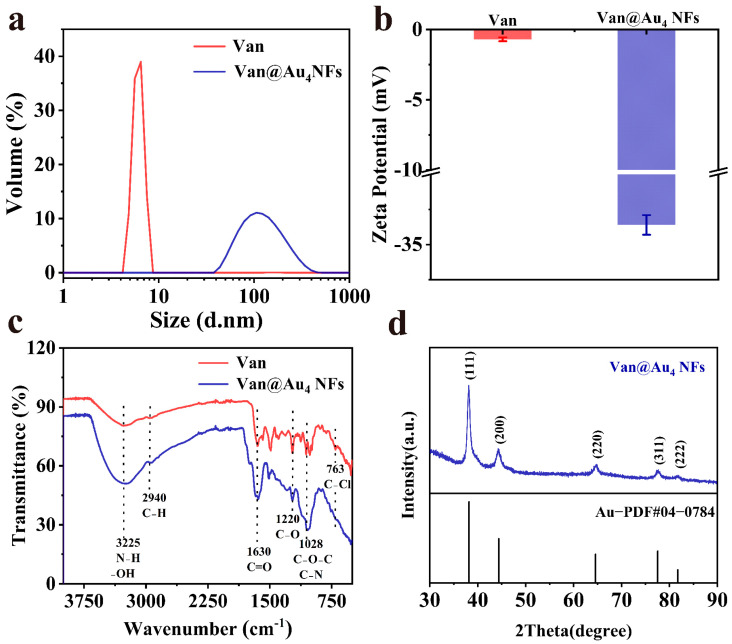
(**a**) Hydrodynamic size of vancomycin hydrochloride and Van@Au_4_ NFs. (**b**) Zeta potential of vancomycin and Van@Au_4_ NFs. (**c**) FTIR of vancomycin and Van@Au_4_ NFs. (**d**) XRD of Van@Au_4_ NFs (JCPDS standard card number: PDF#04-0784 for Au).

Dynamic light scattering was used to measure the hydrodynamic size and zeta potential of Van and Van@Au_4_ NFs. As shown in Figure 6a, the hydrodynamic size of Van@Au_4_ NFs is 122 nm. The hydrodynamic size of Van@Au_4_ NFs is larger than that of Van@Au_4_ NFs measured by TEM. This is due to the different states of Van@Au_4_ NFs during the measurement. DLS measurements show that Van@Au_4_ NFs are hydrated, and the solvent layer formed by Van@Au_4_ NFs in water results in a three-dimensional hydrodynamic size. TEM measurements show that Van@Au_4_ NFs are in a dried state. As shown in Figure 6b, the zeta potentials of Van and Van@Au_4_ NFs are −0.6 mV and −33.6 mV, respectively. Using ascorbic acid to reduce chloroauric acid results in a negatively charged gold colloid surface due to adsorption of acid ions. A larger absolute value of the zeta potential indicates that the Van@Au_4_ NFs are more stable in solution and less prone to aggregation. This shows that peptides are used as templates and stabilizers for the preparation of precious metal nanomaterials, and have the advantages of being easy to chemically modify, having good storage stability without denaturation, and being easy to combine with other strategies or technologies, which is beneficial for peptides to regulate the morphology and size of nanomaterials.

Fourier transform infrared spectroscopy was used to characterize the structural changes of vancomycin hydrochloride before and after the reaction. Figure 6c shows the infrared spectra of Van@Au_4_ NFs and vancomycin. The infrared spectra of Van@Au_4_ NFs and vancomycin exhibit similar peak positions. Van@Au_4_ NFs exhibit absorption peaks at 3225 cm^−1^, 2940 cm^−1^, 1630 cm^−1^, 1220 cm^−1^, 1028 cm^−1^, and 763 cm^−1^. Van@Au_4_ NFs have an absorption peak at 3225 cm^−1^, which is caused by the stretching vibration of N-H of amide and O-H of phenol. Therefore, Van@Au_4_ NFs have amide and hydroxyl groups. The broad peak at 3225 cm^−1^ is blue-shifted to 3280 cm^−1^ in the original vancomycin spectrum; the peak at 2940 cm^−1^ is caused by C-H stretching vibration, which is the same as the peak position on the vancomycin spectrum; the absorption peak at 1630 cm^−1^ is caused by the C=O stretching vibration of amide, which is blue-shifted to 1644 cm^−1^ in the vancomycin spectrum; the absorption peak at 1220 cm^−1^ is caused by the C-O stretching motion of phenol, which is the same as the peak position on the vancomycin spectrum; and the absorption peak at 1028 cm^−1^ is caused by the C-O-C stretching vibration of ester ring ether and the C-N. The absorption peak at 763 cm^−1^ is caused by C-Cl stretching motion, and this peak is red-shifted to 698 cm^−1^ in the vancomycin spectrum. The peak positions of Van@Au_4_ NFs remain essentially unchanged compared to vancomycin, indicating that the chemical form of vancomycin hydrochloride is not significantly altered during the nanoparticle formation process.

To investigate the crystal structure of Van@Au_4_ NFs, X-ray diffractometers were used to characterize the Van@Au_4_ NFs. As shown in Figure 6d, the test scanning angle of Van@Au_4_ NFs is 30°–90°. It can be observed that the characteristic curve of Van@Au_4_ NFs has the five strongest absorption peaks at 38.18°, 44.39°, 64.58°, 77.55° and 81.32°, corresponding to the (111), (200), (220), (311) and (222) crystal planes of Au, respectively. The peaks in Figure 6d are compared with the standard PDF card, which well reflects that the prepared Van@Au_4_ NFs have a face-centered cubic structure.

**Figure 7:**
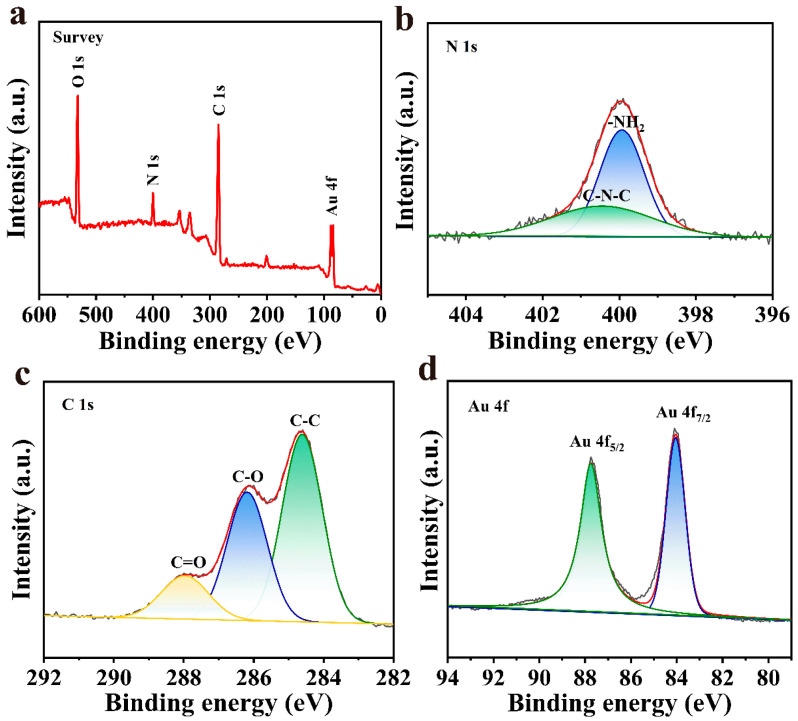
XPS spectra of Van@Au_4_ NFs. (**a**) Full spectrum. (**b**) N 1s. (**c**) C 1s. (**d**) Au 4f.

The elemental composition, chemical state and electronic state of Van@Au_4_ NFs were studied by XPS analysis. As shown in Figure 7a, the characteristic peaks of O, N, C and Au elements are clearly visible in the photoelectron spectrum of the sample. Among them, C, N and O elements are derived from the biological template vancomycin, while the Au element is derived from the precursor HAuCl_4_. The four main peaks at 532.0 eV, 400.0 eV, 284.6 eV, and 84.0 eV correspond to the binding energies of the O 1s, N 1s, C 1s, and Au 4f orbitals on the Van@Au_4_ NFs surface, respectively. Figure 7b shows the N 1s fine spectrum, revealing the characteristic structure of vancomycin: the peak at a binding energy of 399.9 eV is attributed to the sp^2^ hybridized C–N–C bond, while the peak at 400.5 eV corresponds to the primary amino group (–NH_2_). The C 1s spectrum in Figure 7c further confirms the chemical environment of vancomycin, with the peaks at 287.9 eV, 286.2 eV, and 284.6 eV attributed to the C=O, C–O, and C–C structures, respectively [28]. In addition, the Au 4f high-resolution spectrum obtained by narrow area scanning is shown in Figure 7d, which shows two peaks at 87.7 eV and 84.1 eV, corresponding to the typical binding energy of zero-valent gold (Au^0^) [29,30].

### 3.2. Analysis of the Photothermal Performance of Van@Au_4_ NFs

**Figure 8:**
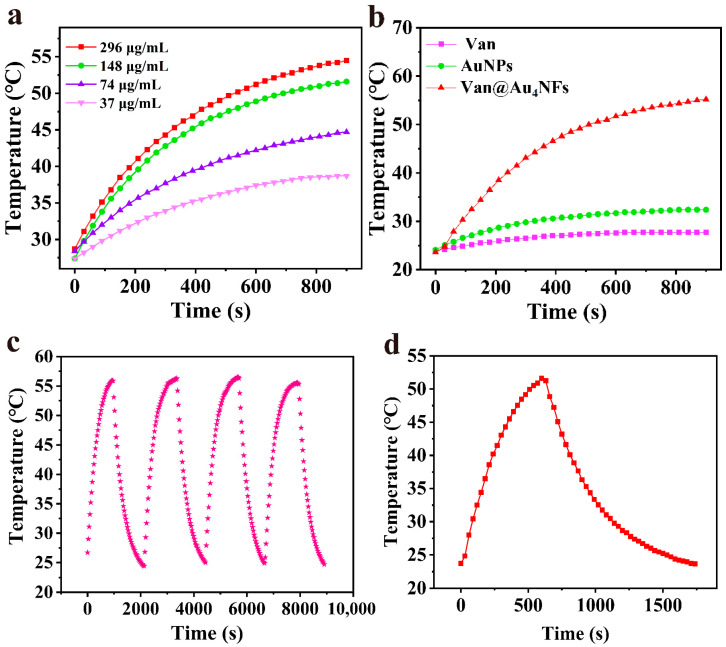
(**a**) Temperature variation of Van@Au_4_ NFs with different concentrations after 808 nm laser irradiation. (**b**) Temperature variation of different samples after 808 nm laser irradiation. (**c**) Four cycles of Van@Au_4_ NFs cooling naturally to room temperature after 808 nm laser irradiation. (**d**) Temperature variation of Van@Au_4_ NFs cooling naturally to room temperature after 808 nm laser irradiation until the temperature stops changing.

Van@Au_4_ NFs exhibit significant absorption at the near-infrared wavelength of 808 nm. Under irradiation with an 808 nm laser (power density of 1.75 W/cm^2^), the gold nanomaterial converts light energy into heat through surface plasmon resonance. To evaluate its in vitro photothermal performance, we tested the temperature rise of Van@Au_4_ NFs at different concentrations under laser irradiation. As shown in Figure 8a, after 10 min of irradiation, the temperatures of samples with concentrations of 296 μg/mL, 148 μg/mL, 74 μg/mL, and 37 μg/mL increased by 22.5 °C, 21.5 °C, 13.8 °C, and 10.0 °C, respectively. The temperature increase accelerated with increasing concentration, reaching a maximum of 51.2 °C at the highest concentration of 296 μg/mL.

To further evaluate the photothermal performance of Van@Au_4_ NFs, pure vancomycin (Van) solution and gold nanoparticle (Au NPs) solution were used as controls and irradiated under the same laser conditions for 10 min. The temperature rises are shown in Figure 8b. The Van solution showed a weak response to the 808 nm laser, with the temperature rising only from room temperature to 27.6 °C; the Au NPs solution temperature increased from 24.1 °C to 31.7 °C. Under the same conditions, the temperature of the Van@Au_4_ NFs reached 51.2 °C, demonstrating superior photothermal conversion performance at the same metal concentration and irradiation time. Furthermore, the photothermal stability of the Van@Au_4_ NFs was investigated through four heating and cooling cycles. As shown in Figure 8c, the sample was able to recover and reach its previous maximum temperature after multiple irradiations, demonstrating excellent thermal stability. Finally, the heating and cooling curves were recorded, as shown in Figure 8d, and the photothermal conversion efficiency of the material was calculated using the Roper equation [31]. Calculations show that the photothermal conversion efficiency of Van@Au_4_ NFs reaches 34.94%, which is significantly higher than reported examples such as gold nanorods (28.7%) [32], Cu_2−x_Se nanocrystals (22%) [33] and gold nanoparticles (21%) [34].

### 3.3. Antibacterial Activity Study

**Figure 9:**
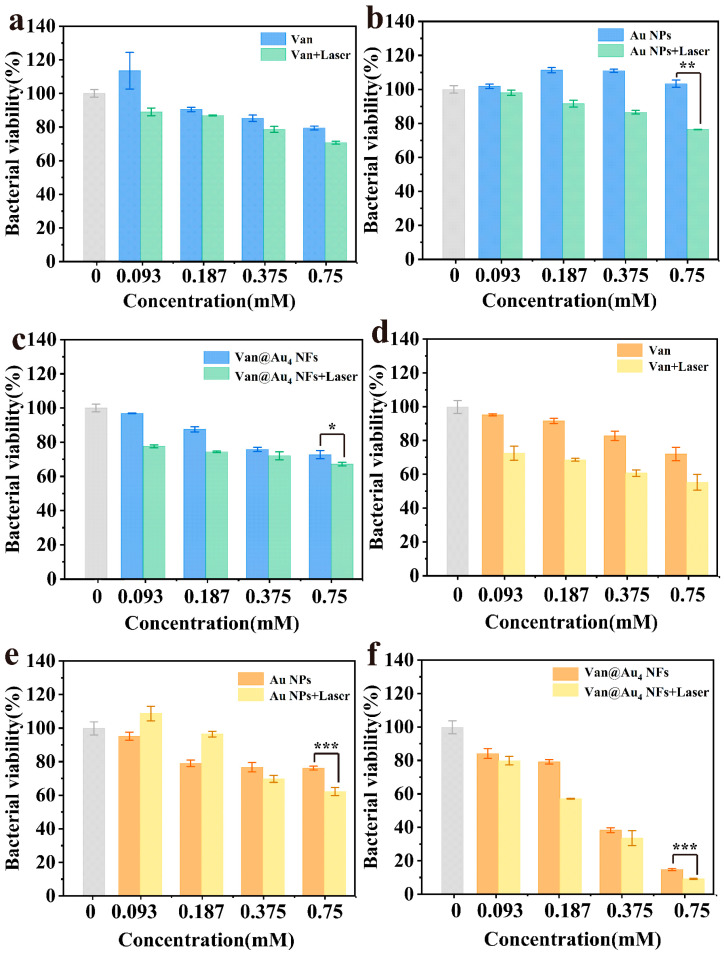
Bacterial activity of *Escherichia coli* after treatment with different concentrations of (**a**) Van, (**b**) Au NPs, and (**c**) Van@Au_4_ NFs and laser. Bacterial activity of *Staphylococcus aureus* after treatment with different concentrations of (**d**) Van, (**e**) Au NPs, and (**f**) Van@Au_4_ NFs and laser (*** *p* < 0.001, ** *p* < 0.01, or * *p* < 0.05).

Vancomycin hydrochloride is a glycopeptide antibiotic that inhibits most Gram-positive bacteria. To test the antibacterial efficacy of Van@Au_4_ NFs, different concentrations of Van@Au_4_ NFs were incubated with bacteria and then irradiated with an 808 nm laser for 10 min to examine their photothermal antibacterial properties. *Escherichia coli* and *Staphylococcus aureus* were used for this study. As shown in Figure 9a–c, at a concentration of 0.75 mM, the *Escherichia coli* activities of the Van + laser group, Au NPs + laser group, and Van@Au_4_ NFs + laser group were 70.69%, 76.40%, and 67.19%, respectively, and the bacterial inhibition rates were 29.31%, 23.60%, and 32.81%, respectively. Compared with the Van + laser group, Van@Au_4_ NFs + laser had a better antibacterial effect on *Staphylococcus aureus*.

Figure 9d–f shows the relative *S. aureus* activity of different samples. At a sample concentration of 0.75 mM, the relative *S. aureus* activity of the Van + laser group, Au NPs + laser group, and Van@Au_4_ NFs + laser group was 55.19%, 62.21%, and 9.20%, respectively, with inhibition rates of 44.81%, 37.79%, and 90.8%, respectively. The bacterial activity of the Van@Au_4_ NFs + laser group was significantly lower than that of the other two groups. As the sample concentration ranged from 0.093 mM to 0.75 mM, the relative *S. aureus* activity of the Van@Au_4_ NFs + laser group was 79.82%, 57.12%, 33.53%, and 9.20%, respectively. As the concentration of Van@Au_4_ NFs increased, its relative bacterial activity decreased. The bacterial activity of the Van@Au_4_ NFs group at a concentration of 0.75 mM was 14.79%, higher than that of the Van@Au_4_ NFs + laser group. These results indicate that Van@Au_4_ NFs exhibited superior antibacterial activity under 808 nm laser irradiation, significantly outperforming the Van group alone.

### 3.4. Cytotoxicity Study

The MTT assay was used to evaluate the inhibitory effects of Van, Au NPs, and Van@Au_4_ NFs on A549 cell viability.

**Figure 10:**
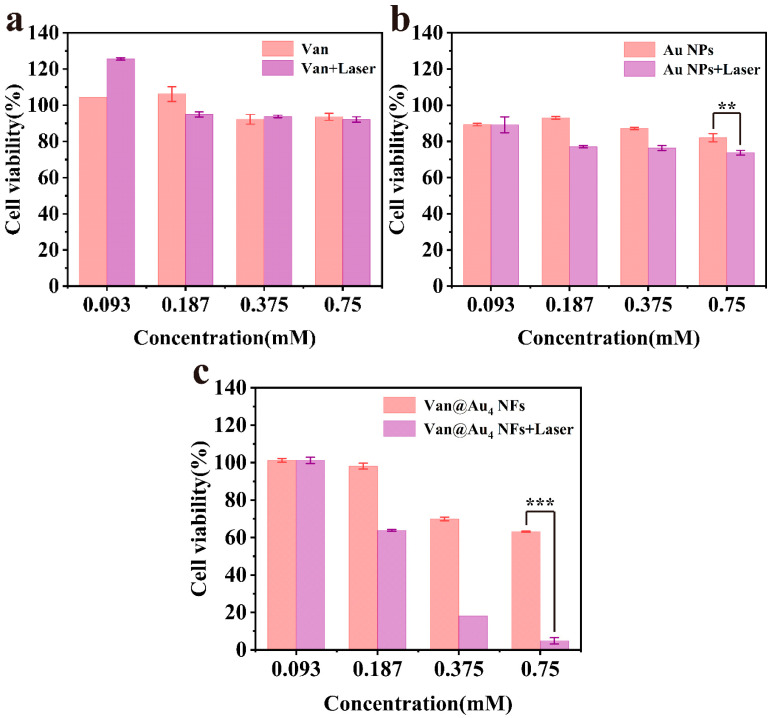
Cell viability of A549 cells after treatment with different concentrations of (**a**) Van, (**b**) Au NPs, and (**c**) Van@Au_4_ NFs plus laser (*** *p* < 0.001, ** *p* < 0.01).

The results are shown in Figure 10a–c. Within the concentration range of 0.093 mM to 0.750 mM, the relative cell viabilities following Van@Au_4_ NFs laser irradiation (Van@Au_4_ NFs + laser group) were 101.21%, 63.79%, 18.15%, and 4.82%, respectively. This indicates that the inhibitory effect on A549 cells gradually increased with increasing nanomaterial concentration. At the highest concentration of 0.75 mM, without near-infrared laser irradiation, the relative cell viabilities of the Van, Au NPs, and Van@Au_4_ NFs groups were 93.56%, 82.08%, and 63.09%, respectively. However, after 808 nm laser irradiation, the cell viabilities of the three groups decreased to 92.14%, 73.75%, and 4.82%, respectively. The results showed that regardless of laser irradiation, neither the Van nor the Au NPs groups exhibited significant tumor inhibition, with cell viability remaining around 70%. In contrast, the Van@Au_4_ NFs group exhibited strong cytotoxicity after near-infrared light irradiation, with the relative viability significantly decreasing to 4.82%, significantly lower than that of the other groups. These results indicate that Van@Au_4_ NFs exhibit remarkable photothermal cytotoxicity against A549 cells.

## 4. Discussion

This study used vancomycin hydrochloride as a multifunctional biological template and a mild reducing agent to prepare gold nanoflower composite Van@Au_4_ NFs through the green biological template method in order to address the dual challenges of tumor treatment and bacterial infection simultaneously. Compared with traditional therapies such as surgery, chemotherapy, radiotherapy, and conventional antibiotics, this photothermal treatment strategy has significant advantages: the introduction of vancomycin avoids the use of toxic surfactants and strong reducing agents in traditional chemical synthesis, which achieves a mild and green preparation process; at the same time, it integrates the bacterial-targeting recognition ability of vancomycin and the photothermal killing ability of gold nanoflower, reducing the non-specific damage caused by traditional chemotherapy drugs. Moreover, near-infrared photothermal treatment has high spatial-temporal controllability, enabling local precise heating and minimizing thermal damage to surrounding normal tissues, providing a new path to solve core problems such as the significant toxic side effects of traditional therapies.

The photothermal performance of gold-based nanomaterials is highly correlated with their morphology, size, and crystal structure. The localized electromagnetic field enhancement effect and the near-infrared absorption range of anisotropic structures are superior to those of spherical nanoparticles. The Van@Au_4_ NFs synthesized in this study exhibit a broad and red-shifted characteristic absorption peak in the 600–900 nm range, with a significantly higher absorption intensity at 808 nm than that of conventional spherical Au NPs. This is attributed to the coordination, reduction, and self-assembly actions of vancomycin guiding the directional growth of gold nanocrystals, forming a flower-like anisotropic structure and changing their surface plasmon resonance mode. Quantitative calculations show that the photothermal conversion efficiency of Van@Au_4_ NFs reaches 34.94%, which is higher than that of typical photothermal materials such as gold nanocages, Cu_2−x_Se nanocrystals, and spherical gold nanoparticles, and this material shows no significant photothermal performance attenuation after 4 laser switching cycles, with good photothermal stability, meeting the basic requirements for repeated treatment. Compared with traditional chemical methods [35] such as sodium citrate reduction and seed growth, the vancomycin template method can achieve directional morphology control, without complex post-treatment, and the prepared nanoflower particles have uniform diameters [36,37]. This method shows significant advantages in photothermal utilization efficiency and preparation operability, providing an effective paradigm for the green and controllable preparation of high-performance anisotropic gold-based photothermal agents.

In vitro experiments confirmed that this system fully combines the Gram-positive bacterial recognition function of vancomycin and the photothermal killing effect of the gold nanoflower, achieving efficient inhibition of Gram-positive bacteria and A549 cells. In the antibacterial experiment, the inhibition rate of Van@Au_4_ NFs combined with the 808 nm laser against *Staphylococcus aureus* was 90.8%, much higher than the simple vancomycin and Au NPs groups, while the antibacterial effect on *Escherichia coli* was limited. This is consistent with the mechanism of vancomycin specifically binding to the D-Ala-D-Ala residues of the Gram-positive bacterial cell wall, confirming that the material retains the targeting recognition characteristics of the template molecule. The antibacterial effect of Van@Au_4_ NFs is due to the specific binding of vancomycin and the local high temperature that leads to bacterial death. Cell experiments showed that without laser irradiation, Van@Au_4_ NFs at the experimental concentration range had no significant toxicity to A549 cells; under near-infrared laser excitation, the material efficiently kills tumor cells through local high heat, reducing the cell survival rate to 4.82%, which is significantly better than the Van and Au NPs control groups. Compared with most single-function nanosystems, the platform constructed in this study can simultaneously address the problems of tumor cell proliferation and secondary bacterial infection, providing a new idea for the treatment of tumors accompanied by infection.

From the perspective of long-term development and clinical translation, the Van@Au_4_ NFs nanosystem possesses significant potential for further optimization. The nanostructure can be precisely tailored by modulating the ratio of vancomycin to chloroauric acid, reaction time, and temperature, thus regulating branch density and particle size distribution to improve photothermal conversion efficiency and in vivo circulation stability. Benefiting from abundant surface active sites, Van@Au_4_ NFs can be further functionalized with targeting peptides, fluorescent probes, or chemotherapeutic drugs to construct multimodal theranostic systems with enhanced tumor accumulation and therapeutic efficacy. This study is currently limited to in vitro antibacterial and antitumor assessments. Future investigations using animal models are warranted to systematically evaluate in vivo distribution, metabolism, inflammatory response, and long-term biosafety for validating in vivo therapeutic performance. Moreover, the vancomycin-mediated green biotemplate strategy can be extended to the controlled synthesis of other noble metal nanomaterials, providing a universal route for developing biocompatible nanomedicines with tunable structures and functions. Overall, this work provides experimental and theoretical support for improving the efficiency of near-infrared photothermal agents, and establishes a foundation for designing multifunctional nanoplatforms for combating Gram-positive bacterial infections and suppressing lung cancer cell proliferation.

## 5. Conclusions

This study used vancomycin as a green template and successfully synthesized gold nanoflowers (Van@Au_4_ NFs) via an environmentally friendly biomineralization method. The synthesis procedure avoided toxic chemical reductants and stabilizers, exhibiting advantages in green synthesis. The as-prepared Van@Au_4_ NFs displayed favorable photothermal properties under 808 nm laser irradiation, with a photothermal conversion efficiency of 34.94% and good cycling stability. In vitro experiments demonstrated that this nanomaterial achieved inhibition rates of 90.8% against *Staphylococcus aureus* and 95.18% against A549 tumor cells, respectively, showing promising in vitro antibacterial and photothermal antitumor activities. This study not only provides a new strategy for the green synthesis of efficient photothermal agents, but also offers experimental support for the in vitro treatment of bacterial infections and the inhibition of tumor cell proliferation.

## Data Availability

The original contributions presented in this study are included in the article.
